# Predictors of competing mortality to invasive breast cancer incidence in the Canadian National Breast Screening study

**DOI:** 10.1186/1471-2407-12-299

**Published:** 2012-07-19

**Authors:** Sharareh Taghipour, Dragan Banjevic, Joanne Fernandes, Anthony B Miller, Neil Montgomery, Andrew K S Jardine, Bart J Harvey

**Affiliations:** 1 Ryerson University, Toronto, ON, M5B 2 K3, Canada; 2 University of Toronto, 5 King's College Road, Toronto, ON, M5S 3 G8, Canada; 3 Dalla Lana School of Public Health, Health Sciences Building, Room 688, 155 College Street, Toronto, ON, M5T 3 M7, Canada

**Keywords:** Invasive breast cancer, Competing mortality, Cancer-specific mortality, Non-cancer mortality, Risk factors

## Abstract

**Background:**

Evaluating the cost-effectiveness of breast cancer screening requires estimates of the absolute risk of breast cancer, which is modified by various risk factors. Breast cancer incidence, and thus mortality, is altered by the occurrence of competing events. More accurate estimates of competing risks should improve the estimation of absolute risk of breast cancer and benefit from breast cancer screening, leading to more effective preventive, diagnostic, and treatment policies. We have previously described the effect of breast cancer risk factors on breast cancer incidence in the presence of competing risks. In this study, we investigate the association of the same risk factors with mortality as a competing event with breast cancer incidence.

**Methods:**

We use data from the Canadian National Breast Screening Study, consisting of two randomized controlled trials, which included data on 39 risk factors for breast cancer. The participants were followed up for the incidence of breast cancer and mortality due to breast cancer and other causes. We stratified all-cause mortality into death from other types of cancer and death from non-cancer causes. We conducted separate analyses for cause-specific mortalities.

**Results:**

We found that “age at entry” is a significant factor for all-cause mortality, and cancer-specific and non-cancer mortality. “Menstruation length” and “number of live births” are significant factors for all-cause mortality, and cancer-specific mortality. “Ever noted lumps in right/left breasts” is a factor associated with all-cause mortality, and non-cancer mortality.

**Conclusions:**

For proper estimation of absolute risk of the main event of interest common risk factors associated with competing events should be identified and considered.

## Background

Women in a clinical trial with breast cancer incidence as the endpoint may die due to causes other than breast cancer before the occurrence of the cancer. Competing events should be taken into account in evaluating the efficacy and cost-effectiveness of screening interventions both at the population level and for a given individual (personalized screening regimes). A specific screening intervention may be recommended for a woman based on her age and other characteristics, such as having a family history of breast cancer. However, these characteristics can also affect the occurrence of competing events; so, it is essential to study the effect of risk factors on both breast cancer incidence and its competing events such as mortality due to other causes.

Some studies have focused on the main event of interest and considered competing risks. Fang et al. [1] examined the potential role of nonsteroidal anti-inflammatory drugs on prostate cancer specific mortality while controlling for other competing causes of death. Yi et al. [2] determined the factors associated with a contralateral prophylactic mastectomy while taking into account the competing risk of the recurrence of the primary breast cancer. Mell et al. [3] identified predictors of non-cancer causes of death in head and neck cancer and developed a risk stratification model for these competing events. Mell et al. [4] used competing risk modeling to identify predictors of non-cancer mortality in women with early breast cancer, while considering disease recurrences as competing risks.

Other studies focused on all competing events and investigated the effect of some risk factors on the main event of interest and its competing risks. Several studies used cohort life tables to derive probability estimates for death due to breast cancer as well as mortality due to other causes. Hence, they were able to estimate reductions in breast cancer mortality [5,6]. Lambert et al. [7] estimated and partitioned the crude probability of all-cause mortality to the probabilities due to cancer and other causes. Crude probabilities can be used to understand the impact of disease on individual patients and help assess different treatment options. Daskivich et al. [8] assessed the competing risks of mortality from non-prostate cancer on patients with prostate cancer. Barnes et al. [9] emphasized that some risk factors associated with breast cancer have been shown to affect the risk of other health outcomes, so competing risks and benefits may influence public health policy decisions. Vilaprinyo et al. [6] estimated the risk of death from causes other than breast cancer; used for assessing the effect of mammography screening on breast cancer mortality.

**Figure 1:**
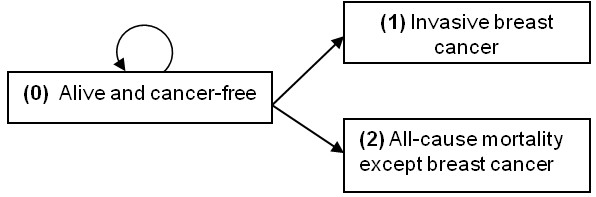
Schematic illustration of the three terminating points in the study.

In another study [10], we have investigated the association of 39 risk factors with breast cancer incidence in the presence of competing risk. We used data from the Canadian National Breast Screening Study (CNBSS) in which the information on risk factors were collected at enrolment or recorded by a nurse or physician at the initial physical examination of the breasts. In the CNBSS, women were enrolled alive and had to be cancer-free. They were randomly assigned to study and control groups and were followed up for breast cancer incidence and mortality. By the end of the study period, a woman might have been diagnosed with breast cancer, died from any causes including breast cancer, or remained alive and cancer-free. A schematic illustration of the three possible terminating points is shown in Figure 1.

The main focus of the current study and of [10] is on breast cancer incidence and its risk prediction model. Therefore, in these two studies, we terminate the follow-up of a woman as soon as she is diagnosed with breast cancer. Thus, we do not study the progression of a tumor from diagnosis to death.

Breast cancer incidence and mortality due to non-breast cancer causes are competing risks and both should be analyzed. The necessity of studying both events is given in detail in Discussion section of the paper. In this paper, we investigate the effect of 39 risk factors on mortality due to causes other than breast cancer. So, the current paper and [10] are complimentary. We identify the factors common to both breast cancer incidence and competing mortality. In addition, we stratify all-cause mortality (excluding breast cancer), comparing it to death caused by other types of cancer and death from non-cancer causes. We conduct separate analyses for all-cause mortality and the two cause-specific mortalities. The reason for stratification is to examine whether any of the risk factors are particularly associated with both breast cancer incidence and cancer mortality. The findings in this case are more informative and help us understand better the biological mechanism through which a risk factor influences cancer and non-cancer mortality.

## Methods

### Study Population and Period

**Figure 2:**
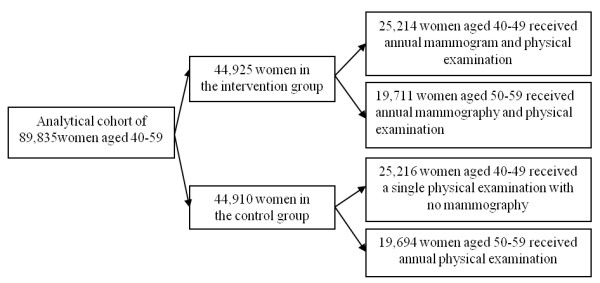
A flow diagram of the CNBSS.

The Canadian National Breast Screening Study (CNBSS) has been described previously [10-12]. Its main objective is to assess the effect of mammography in reducing breast cancer mortality. The study consists of two randomized controlled trials of 89,835 women; 50,430 aged 40–49 and 39,405 aged 50–59. The women were recruited at 15 Canadian centres between 1980 and 1985. All participants signed an informed consent form developed with approval from the University of Toronto Human Experimentation Committee when they enrolled in the CNBSS which included explicit agreement for linkage to vital statistics records and analysis of the data in the future. Women in the age group 40–49 were randomly selected to receive either an annual mammogram and physical examination (intervention group) or only an initial physical examination with no mammography (control group). Women aged 50–59 were randomly selected to receive either an annual mammogram and physical examination (intervention group) or an annual physical examination only (control group). A flow diagram of the CNBSS is presented in Figure 2.

Woman in the CNBSS were not pregnant at the time of enrolment, had no history of breast cancer and no mammogram in the past 12 months. At enrolment, they completed enrolment and epidemiology questionnaires which included information on demographics, life style, family history of breast cancer, and personal history of breast disease. Moreover, at enrolment a nurse or physician performed a physical examination of the breasts and recorded information on several risk factors.

The CNBSS contains information on breast cancer diagnoses reported for women in the intervention group; these women received up to five mammograms. In addition, breast cancer diagnoses were identified by record linkage with the National Cancer Registry and deaths through linkage with the Canadian Mortality Database at Statistics Canada for both the control and the intervention groups after the annual screenings had ended.

For this analysis we consider 1980–1989 as the study period and exclude breast cancers which were diagnosed less than six months after enrolment to eliminate long-term prevalent breast cancers.

A total of 89,434 women were considered in our study: of these, 944 were diagnosed with invasive breast cancer, 922 died from causes other than breast cancer (Table 1 describes the causes of death for these 922 women), and 87,568 were neither diagnosed with breast cancer nor had died from other causes by the end of 1989. Of the 922 who died from causes other than breast cancer, 536 died from cancers other than breast cancer, and 386 deaths were due to non-cancer causes.

**Table 1:** Causes of deaths

**Death Cause**	**# Of Cases (n = 922)**
*Other cancers (excluding breast)*	(n = 536)
- Lung cancer	126
- Colon cancer	64
- Ovarian cancer	59
- Pancreatic cancer	48
- Other sites	239
*Non cancer deaths*	(n = 386)
- Infections and Parasitic Diseases	5
- Endocrine, nutritional and metabolic diseases and immunity disorders	18
- Diseases of blood and blood forming organs	1
- Diseases of nervous system and sense organs	17
- Diseases of the circulatory system	183
- Diseases of the respiratory system	18
- Diseases of the digestive system	27
- Diseases of the genitourinary system	3
- Diseases of the musculoskeletal system and connective tissue	3
- Congenital anomalies	3
- Symptoms, signs and ill-defined conditions	7
- Injury and poisoning	25
- Factors influencing health status	1 (allergy)
- External causes	74
- Unknown	1

### Risk Factors and Data Preparation

This study considers 39 risk factors (see Tables 2, 3,4, 5) collected at enrolment or recorded by a nurse or physician at the initial physical examination of the breasts. We classify these factors into four groups: socio-demographic factors, reproductive factors, lifestyle and health behaviours, and history of breast disease. For a premenopausal woman, “menstruation length” is the difference between her “age at entry” and “age at menarche”, and for a postmenopausal woman, it is the difference between her “age at the last menstrual period” and “age at menarche”. Less than 5 % of the values of all data were missing and have been imputed. A normal linear regression model is used to impute missing values in the continuous variables. Missing values in the categorical variables are imputed using a logistic or generalized logistic model. Each woman is given a score for families/relatives with breast cancer. Each relative, depending on her degree (first, second, third, fourth, fifth degree and above) makes a contribution of $2^{\left(6-\text{degree number}\right)}$ to the score value. For example, a woman with one first degree and one second degree relative with breast cancer is given a score of $2^{\left(6-1\right)}+2^{\left(6-2\right)}=48$. A woman with no relatives with breast cancer receives a score of 1 [10]. The calculation method for score value is adapted from the U.S. Preventive Services Task Force recommendations on genetic risk assessment and BRCA mutation testing for breast and ovarian cancer susceptibility [13].

**Table 2:** Characteristics of the study cohort: Socio-demographic factors

**Variable**	**n = 89434(%)**
Age at entry (mean±SD)	48.51(±5.61)
Birth place	
Canada	73611 (82.31)
Foreign	15823 (17.69)
Years in Canada	
Born in Canada	73538 (82.22)
1-5	516 (0.58)
6-10	955 (1.07)
11-20	4567 (5.11)
> 20	9858 (11.02)
Mother's birth place	
Canada	59103 (66.09)
Foreign	30331 (33.91)
Father's birth place	
Canada	56169 (62.80)
Foreign	33265 (37.20)
Ethnic origin	
Canada	336 (0.38)
Foreign	89098 (99.62)
Marital status	
Ever Married	84046 (93.98)
Never Married	5388 (6.02)
Occupations	
Homemakers	31186 (34.86)
Clerical Occupations	18922 (21.16)
Social sciences, religion, teaching, artistic, literary and related	9808 (10.97)
Medicine and health	8405 (9.40)
Administrative and Managerial	6026 (6.74)
Sales Occupations	4836 (5.41)
Service Occupations-food and personal service	3460 (3.87)
Processing, machining, fabricating, and construction occupations	1187 (1.33)
Natural sciences, engineering and math	684 (0.76)
Transport, material handling, equipment operating	622 (0.70)
Farming, forestry and fishing	567 (0.63)
Others	3731 (4.17)
Allocation group	
No mammography	44744 (50.03)
Mammography	44690 (49.97)

**Table 3:** Characteristics of the study cohort: Reproductive factors

**Variable**	**n = 89434(%)**
Age at menarche	
9-10	2675 (2.99)
11-13	59433 (66.46)
14-16	26231 (29.33)
17-18	1095 (1.22)
Still have periods	
Yes	39281 (43.92)
No	50153 (56.08)
Menstrual status	
Pre-menopause	49201 (55.01)
Post-menopause	40233 (44.99)
Menstruation length (years) (mean±SD)	32.10(±5.85)
Number of live births	
Nulliparous	13173 (14.73)
1-2	32781 (36.65)
3-4	33952 (37.96)
> = 5	9528 (10.66)
Regular periods	
Yes	70635 (78.98)
No	18799 (21.02)
Had a hysterectomy	
Yes	27584 (30.84)
No	27584 (69.16)
Had a bi-lateral oophorectomy	
Yes	9567 (10.70)
No	79867 (89.30)
Ever pregnant	
Yes	78870 (88.19)
No	10564 (11.81)
Number of pregnancies	
0	13173 (14.73)
1-2	23050 (25.77)
3-4	34721 (38.82)
5-10	17966 (20.09)
11-17	524 (0.59)
Pregnancies lasted < 4 months	
0	60228 (67.35)
1-2	25563 (28.58)
3-4	2998 (3.35)
5-10	645 (0.72)
Age at first child birth	
N/A	13173 (14.73)
14-19	8345 (9.33)
20-25	42234 (47.22)
26-30	18756 (20.97)
31-34	4683 (5.24)
35-39	2011 (2.25)
40-49	232 (0.26)

**Table 4:** Characteristics of the study cohort: Lifestyle and health behaviours

**Variable**	**n = 89434(%)**
***Lifestyle and health behaviours***	
Practice BSE	
Yes	44802 (50.10)
No	44632 (49.90)
Ever used oral contraceptives	
Yes	53694 (60.04)
No	35740 (39.96)
Length of oral contraceptive used (months) (mean±SD)	32.70(±46.98)
Ever used estrogen, w/wo progesterone	
Yes	24489 (27.38)
No	64945 (72.62)
Length of estrogens/progesterone used (months) (mean±SD)	13.11(±35.67)
Ever smoked	
Yes	45091 (50.42)
No	44343 (49.58)
Cigarettes per day	
0 per day	44343 (49.58)
Occasionally	1907 (2.13)
1-5 per day	6981 (7.81)
6-10 per day	9023 (10.09)
11-25 per day	21963 (24.56)
26-50 per day	4939 (5.52)
>50 per day	278 (0.31)
Ever had a mammogram	
Yes	24429 (27.32)
No	65005 (72.68)
Number of mammograms	
0	65088 (72.78)
1	16324 (18.25)
2-3	6610 (7.39)
>4	1412 (1.58)

**Table 5:** Characteristics of the study cohort: history of breast disease

**Variable**	**n = 89434(%)**
Ever been told to have breast cancer	
Yes	115 (0.13)
No	89319 (99.87)
Ever noted lumps in breasts	
Yes	5139 (5.75)
No	84295 (94.25)
Ever noted pain in breasts	
Yes	14951 (16.72)
No	74483 (83.28)
Ever noted discharge from breasts	
Yes	1881 (2.10)
No	87553 (97.90)
Families with breast cancer score (mean±SD)	14.77(±21.22)
Ever have/had other types of breast disease	
Yes	14363 (16.06)
No	75071 (83.94)
Abnormality in left breast found by nurse	
Yes	14769 (16.51)
No	74665 (83.49)
Abnormality in right breast found by nurse	
Yes	13748 (15.37)
No	75686 (84.63)
Female relatives with breast cancer	
Yes	31574 (35.30)
No	57860 (64.70)

### Statistical Method

In this study, we use multivariate cause-specific Cox regression analysis to investigate the associations of the 39 risk factors with all-cause mortality, cancer-specific mortality, and non-cancer mortality. Incidence of breast cancer is considered a competing event in the analysis of all-cause mortality. In our analysis of the cause-specific mortality from cancer and non-cancer causes, mutually exclusive events are the competing events. For example, breast cancer incidence and non-cancer mortality are considered events competing with cancer-specific mortality.

It should be noted that hazard of subdistribution proposed by Fine and Gray [14] is another method for regression modeling of an event in the presence of competing risks. The hazard of subdistribution can be used for directly modeling the effect of covariates on the cumulative risk of the event of interest. However, a cause-specific model must be used when the goal is to investigate the biological effect of risk factors on the event of interest [15]. We also fitted the Fine-Gray model to our data and obtained very similar results to those obtained from the cause-specific hazard model. Results from the hazard of subdistribution model are not shown in this paper due to space limitations. We use the procedure PHREG in SAS v. 9.3 (SAS Institute, Cary, NC) to build regression models. To construct the models we first conduct univariate analysis of each individual variable, we then combine the significant factors in a multivariate model to adjust the risk for all significant factors. Moreover, we perform a backward model selection to recheck the final model. We considered a variable (risk factor) statistically significant if its probability (*P*) value was less than 0.05.

We checked the interaction of the variables with each other and with time to event (time of death or censoring time) to find those variables with a time-varying effect on the risk of mortality. No interaction terms were found to be statistically significant.

**Figure 3:**
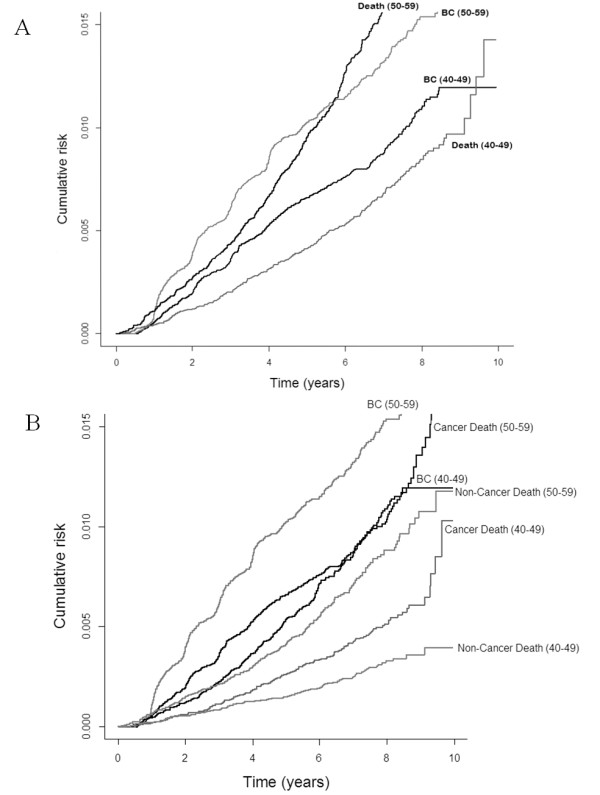
**Ten-year cumulative risks by age groups.** A, invasive breast cancer and the all-cause mortality. B, invasive breast cancer, the all-cause mortality, cancer-specific and non-cancer mortalities.

In Figure 3, we used a non-parametric method formulated by Kalbfleisch and Prentice [16] to estimate cause-specific hazard and obtained a discrete estimate of the cumulative incidence function (cumulative risk). For this purpose, we used function *cuminc* in package *cmprsk* in R ( http://cran.r-project.org/web/packages/cmprsk/cmprsk.pdf).

## Results

From 89,434 participants in the study, 1.06% (N = 944) were diagnosed with invasive breast cancer, 1.03% (N = 922) died from causes other than breast cancer, and 97.9 % (N = 87,568) were alive and not diagnosed with breast cancer by the end of 1989. 58.1% (N = 536) of the deaths were due to other types of cancer, and 41.9% (N = 386) due to causes other than cancer. The mean times from enrolment to cancer diagnosis and to death are 3.52 (SD 2.0) and 4.26 (SD 2.17) years respectively. The median from enrolment to cancer diagnosis and to death are respectively 3.18 and 4.23 years.

The mean and median follow-up time of women in the censored group (alive and cancer-free by the end of the study) are 6.72 (SD 1.32) and 6.39 years, respectively. Figure 3.A shows the observed ten-year cumulative risk of invasive breast cancer and all-cause mortality (excluding breast cancer death) stratified by two age-groups 40–49 and 50–59. The risk of breast cancer incidence is always higher than the risk of all-cause mortality for younger women (40–49) within the follow-up period. However, for older women (50–59), although breast cancer incidence is lower than the risk of death in about the first six years of follow-up; the curves for breast cancer incidence and all-cause mortality cross at this point and the probability of death is higher than breast cancer incidence subsequently.

Figure 3.B shows the ten-year cumulative risk of breast cancer incidence, cancer-specific mortality, and non-cancer mortality stratified by the two age-groups. When we compare the breast cancer and mortality curves in both age groups, we see that the women in our study always have a higher probability of breast cancer incidence than cancer-specific and non-cancer mortality. Moreover, although for women aged 40–49 the risk of death from cancer and non-cancer causes is similar in the first two and half years of follow-up, death from cancer is more likely to occur than death from non-cancer causes afterwards. For women in the age-group 50–59, death from cancer is more likely to occur than non-cancer mortality in the entire follow-up period. The probability estimates and their standard errors for breast cancer incidence, cancer mortality, and non-cancer mortality at eight years after follow-up are 0.013 (0.00047), 0.007 (0.00037), and 0.006 (0.00033) respectively, for the overall population.

### Predictors of All-cause Mortality

Table 6 presents the factors found to be statistically significant for all-cause mortality. The reference levels of the categorical variables are also shown in the table. As an example, “1-2 children” is the reference level for “number of live births”. For continuous variables, such as age or menstruation length, the hazard ratio is the change in the hazard for a one-unit increase in the value of the variable, holding all other variables constant. The results show that older women have higher risk of all-cause mortality (HR 1.12, 95% CI 1.10-1.13). For example, compared to a 40-year old woman with the same characteristics, a woman aged 50 at enrolment is almost three times ( $1.118^{10}\approx 3.00$) more likely to die due to any causes at any given point in time. Taking estrogen or progesterone supplements for a longer time and having more years of menstruation are slightly protective factors against all-cause mortality. There is no evidence that a nulliparous woman is more likely to die due to any causes than a woman with one or two live births; however, having more than two live births decreases the probability of all-cause mortality (HR 0.84 and 0.76 for three-four and more than four live births, respectively). Breast self examination (BSE) is another protective factor for all cause mortality (HR 0.83, 95% CI 0.73-0.94). Although smoking less than 11 cigarettes per day does not statistically significantly increase all-cause mortality, smoking 11 or more cigarettes per day significantly increases the probability of death due to any causes. For example, a woman who smokes more than 50 cigarettes per day is four times more at risk of death than a non-smoker.

**Table 6:** Adjusted risk for all statistically significant factors for all-cause mortality excluding breast cancer death

**Variable**	**Parameter Estimate**	**HR (95% CI)**	***P-value***
Age at entry	0.11115	1.12 (1.10-1.13)	<.0001
Length of estrogen/progesterone used (months**)**	−0.00285	0.997 (0.995-0.999)	0.0019
Menstruation length (years)	−0.01088	0.989 (0.979-1.000)	0.0458
Had a hysterectomy (Ref^*^: No) Had a hysterectomy (Ref^*^: No)			
Yes	0.19090	1.21 (1.04-1.41)	0.0153
Number of live births (Ref: 1–2)			0.0282
Nulliparous	−0.00203	0.998 (0.810-1.230)	0.9848
3-4	−0.17410		0.0300
> = 5	−0.27340	0.84 (0.72-0.98) 0.76 (0.61-0.95)	0.0180
Breast self examination (BSE) practise (Ref: No)			
Yes	−0.18797	0.83 (0.73-0.94)	0.0048
Cigarettes smoked per day (Ref: 0 per day)			<.0001
Occasionally	0.18753	1.21 (0.76-1.91)	
1-5 per day	0.11747	1.13 (0.86-1.48)	0.4257
6-10 per day	0.04960	1.05(0.82-1.35)	0.3996
11-25 per day	0.55745	1.75 (1.50-2.04)	0.6960
26-50 per day	1.01296	2.75 (2.21-3.43)	<.0001
>50 per day	1.39405	4.03 (2.08-7.82)	
			<.0001
			<.0001
Ever noted lumps in breasts (Ref: No)			
Yes	0.28345	1.33 (1.02-1.72)	0.0333
Ever noted discharge from breasts (Ref: No)			
Yes	0.39762	1.49 (1.01-2.19)	0.0430
Age at first child birth (Ref: 20–25)			0.0053
14-19	0.34338	1.41 (1.14-1.74)	0.0016
26-30	−0.10960	0.90 (0.75-1.07)	0.2284
31-34	−0.08054	0.92 (0.68-1.25)	0.6017
35-39	−0.29037	0.75 (0.46-1.21)	0.2351
40-49	−1.16225	0.31 (0.04-2.23)	0.2463

Ref^*^: Reference level.

Women who have ever noted lumps in their breasts or have had discharge from their breasts have respectively 1.3 and 1.5 times higher risk of death than those who have not. Having the first child at age 14 to 19 is associated with a 1.4-fold higher risk of death (HR 1.41, 95% CI 1.14-1.74) than having the first child at age 20 to 25.

### Predictors of Cancer-specific Mortality

The predictors for mortality due to cancer (excluding breast cancer) are presented in Table 7. “Age at entry”, “length of estrogen/progesterone used”, “menstruation length”, “breast self examination”, and “having the first child birth at age 14-19” are factors having a similar or the same effect on the risk of all-cause mortality and mortality due to cancer. Women who have a bi-lateral oophorectomy are 1.3 times more at risk of death due to cancer than those who do not. As for all-cause mortality, there is no evidence that a nulliparous woman is more likely to die due to cancer than a woman with one or two live births, but having more than two live births decreases the probability of cancer death (HR 0.74 and 0.46, respectively for 3–4 and > = 5 live births). There is evidence that a woman with more than 10 pregnancies has a 3.3-fold greater risk of cancer death. Women who smoke more than 10 cigarettes per day have a higher risk of cancer mortality (HR 1.72, 2.90, and 2.78, respectively, for smoking 11–25, 26–50, and > 50 cigarettes per day).

**Table 7:** Adjusted risk for all statistically significant factors for cancer-specific mortality

**Variable**	**Parameter Estimate**	**HR (95% CI)**	***P***
Age at entry	0.10405	1.11 (1.09-1.13)	<.0001
Length of estrogen/progesterone used (months**)**	−0.00308	0.997 (0.995-0.999)	0.0132
Menstruation length (years)	−0.01369	0.986 (0.974-0.999)	0.0406
Had a bi-lateral oophorectomy (Ref^*^: No)			
Yes	0.29487	1.34 (1.05-1.72)	0.0206
Number of live births (Ref: 1–2)			0.0062
Nulliparous	−0.08554	0.92 (0.69-1.22)	0.5574
3-4	−0.30281	0.74 (0.56-0.98)	0.0364
> = 5	−0.77717	0.46 (0.30-0.71)	0.0005
Breast self examination (BSE) practise (Ref: No)			
Yes	−0.17698	0.84 (0.71-0.94)	0.0424
Cigarettes smoked per day (Ref: 0 per day)			<.0001
Occasionally	0.24987	1.28 (0.72-2.30)	0.3998
1-5 per day	−0.01604	0.98 (0.68-1.44)	0.9335
6-10 per day	−0.10999	0.90 (0.63-1.27)	0.5326
11-25 per day	0.54201	1.72 (1.41-2.10)	<.0001
26-50 per day	1.06327	2.90 (2.19-3.83)	<.0001
>50 per day	1.02201	2.78 (1.03-7.51)	0.0438
Number of pregnancies (Ref: 1–2)			0.0136
3-4	0.04212	1.04 (0.78-1.40)	0.7812
5-10	0.20064	1.22 (0.84-1.78)	0.2913
11-17	1.19964	3.32 (1.58-6.97)	0.0015
Age at first child birth (Ref: 20–25)			0.0185
14-19	0.33392	1.40 (1.06-1.84)	0.0185
26-30	−0.21654	0.81 (0.64-1.02)	0.0741
31-34	−0.28472	0.75 (0.50-1.14)	0.1824
35-39	−0.32799	0.72 (0.39-1.33)	0.2943
40-49	−0.68653	0.50 (0.07-3.60)	0.4942

Ref^*^: Reference level.

### Predictors of Non-cancer Mortality

Table 8 presents the factors found to be statistically significant for non-cancer mortality. The table shows that “age at entry” and “length of estrogen/progesterone used” have similar effects on both non-cancer mortality and all-cause mortality. In addition, women whose ethnic origin was reported as not being Canadian have a 5-fold (HR 0.22, 95% CI 0.10-0.46) lower risk of non-cancer mortality than women whose ethnic origin was reported as Canadian. Women who have had a hysterectomy are 1.3 times more likely to die from non-cancer causes than those who have not. Women who reported smoking more than 10 cigarettes per day were found to have a higher risk of non-cancer mortality (HR 1.82, 2.62, and 5.86, respectively, for smoking 11–25, 26–50, and > 50 cigarettes per day). Finally, women who have ever noted lumps in their breasts or have had discharge from their breasts have respectively 1.6 and 2.0 times more risk of non-cancer death than those who have not.

**Table 8:** Adjusted risk for all statistically significant factors for non-cancer mortality

**Variable**	**Parameter Estimate**	**HR (95% CI)**	***P-value***
Age at entry	0.11412	1.12 (1.10-1.14)	<.0001
Length of estrogen/progesterone used (months**)**	−0.00293	0.997 (0.994-1.000)	0.0398
Ethnic origin (Ref^*^: Canada)			
Foreign	−1.51760	0.22 (0.10-0.46)	<.0001
Had a hysterectomy (Ref: No)			
Yes	0.28427	1.33 (1.07-1.65)	0.0093
Cigarettes smoked per day (Ref: 0 per day)			<.0001
Occasionally	0.08311	1.09 (0.51-2.32)	
1-5 per day	0.27274	1.31 (0.88-1.96)	0.8298
6-10 per day	0.23232	1.26 (0.88-1.81)	0.1789
11-25 per day	0.59759	1.82 (1.43-2.31)	0.2057
26-50 per day	0.96370	2.62 (1.83-3.75)	<.0001
>50 per day	1.76857	5.86 (2.40-14.30)	
			<.0001
			0.0001
Ever noted lumps in breasts (Ref: No)			
Yes	0.49413	1.64 (1.14-2.36)	0.0080
Ever noted discharge from breasts (Ref: No)			
Yes	0.70502	2.02 (1.20-3.40)	0.0079

Ref^*^: Reference level.

Table 9 compares the significant factors in all three mortality models and the model for breast cancer incidence [10] (factors for breast cancer incidence are reported in detail in [10]). “Age at entry”, “length of estrogen/progesterone used (months**)**”, and “cigarettes smoked per day” are common to the three morality models. If we compare the significant risk factors for mortality with those associated with breast cancer incidence, we find “age at entry” appears in all four models. “Menstruation length” and “number of live births” are statistically significant risk factors for breast cancer incidence, all-cause mortality, and cancer-specific mortality. “Ever noted lumps in breasts” is associated with breast cancer incidence, all-cause mortality, and non-cancer mortality.

**Table 9:** Comparing the predictors of breast cancer incidence, all-cause mortality, cancer-specific mortality and non-cancer mortality

**Variable**	**Breast cancer incidence**	**All-cause mortality**	**Cancer mortality**	**Non-cancer mortality**
Age at entry	**+**	**+**	**+**	**+**
Interaction of age at entry and time to event	**+**	**-**	**-**	**-**
Ethnic origin (Foreign)	**-**	**-**	**-**	**+**
Length of estrogen/progesterone used (months**)**	**-**	**+**	**+**	**+**
Menstruation length (years)	**+**	**+**	**+**	**-**
Had a hysterectomy	**-**	**+**	**-**	**+**
Had a bi-lateral oophorectomy	**-**	**-**	**+**	**-**
Number of live births	**+**	**+**	**+**	**-**
Interaction of nulliparous level and time to event	**+**	**-**	**-**	**-**
Breast Self examination (BSE) practise	**-**	**+**	**+**	**-**
Cigarettes smoked per day	**-**	**+**	**+**	**+**
Ever noted lumps in breasts	**+**	**+**	**-**	**+**
Interaction of lumps and time to event	**+**	**-**	**-**	**-**
Ever noted discharge from breasts	**-**	**+**	**-**	**+**
Ever have/had other types of breast disease	**+**	**-**	**-**	**-**
Abnormality in left breast found by nurse	**+**	**-**	**-**	**-**
Age at first child birth	**-**	**+**	**+**	**-**
Number of pregnancies	**-**	**-**	**+**	**-**
Families with breast cancer score	**+**	**-**	**-**	**-**
**+** indicates the factor is significant				
**-** indicates the factor is not significant				

## Discussion

In this study, we identify the predictors for all-cause mortality, cancer-specific mortality (excluding mortality from breast cancer), and non-cancer mortality. As previously reported, mortality due to causes other than breast cancer is a competing risk for breast cancer incidence in the data from the Canadian National Breast Screening Study (CNBSS). It should be noted that we investigated the associations for allocation group (no-mammography vs. mammography) to both breast cancer incidence and its competing mortality. We conducted univariate and multivariate analyses, and in none of the models, did this categorical variable reach statistical significance when combined with other risk factors. This does not mean there is no some real difference in breast cancer incidence in these two groups, but the data do not show it statistically. Otherwise, the analysis would have been stratified by allocation group and separate results obtained for each group. Therefore, to make use of a larger sample size for statistical analysis we combined all the data without stratification by allocation group.

We found that using estrogen or progesterone supplements for a longer time and longer menstruation are both protective factors against all-cause mortality. These results are consistent with other studies’ findings that mortality among women who use hormones is lower than among nonusers [17,18], and the later the onset of menopause, the longer a woman is likely to live [19,20]. A negative association of these two factors with mortality could primarily be explained by reducing the occurrence of cardiovascular disease [18,19].

The all-cause mortality analysis revealed a decrease in mortality with number of live births. In the literature, there is no consistent pattern of mortality with the number of births [21]. Some studies show a clear decrease in mortality for women with 2–3 children compared to nulliparous women, and a non-statistically significant increase in the risk for women with more than three children [22]. The result of some other studies is consistent with our result and shows an overall negative association between higher parity and mortality [23].

Similar to our result which shows an increase in the risk of mortality when a woman has her first child at a younger age (14–19), Grundy and Kravdal [23] also found higher mortality for parents who had their first child at a younger age which tended to decrease with older age at first birth.

Similar to the results from all-cause mortality, increase in the number of live-births exhibits a decreasing trend in the risk of cancer-specific mortality, although the nulliparous level does not reach statistical significance. As the number of pregnancies increases the risk of cancer-specific mortality increases as well, although only more than ten times of pregnancy reaches statistical significance. Moreover, increasing age at first child birth shows a clear decreasing trend for risk of cancer-specific mortality; however, only decrease in the risk of women who had their first child-birth at age 20–25 compared to those who had it at age 14–19 is statistically significant.

We also found that women who were practicing BSE on enrolment also had lower all-cause mortality. Arguably, women who practice BSE are more likely to have better personal health practices such as exercise, nutrition, etc.

Event though we observe no trend in the first three levels of the number of cigarettes smoked per day (i.e. up to 11–25 cigarettes per day), the next three levels clearly show an increasing trend in the risk in all three mortality models.

Ever noting lumps in the breasts or having discharge from the breasts are both associated with non-cancer mortality. Although we do not have sufficient data to investigate biological association of these two factors with each cause of mortality, one reason for increased risk of non-cancer mortality could be anxiety caused by discovery of a breast abnormality [24]. Women who experience the discovery will be coping with fears about the future and dealing with emotional ups and downs [24].

By using the CNBSS, our study takes advantage of a large cohort linked to the Canadian Cancer Registry and the Mortality Database at Statistics Canada and relatively complete data collected at the time of enrolment and the initial physical examination of the breasts. This makes our investigation of the association of 39 different risk factors with breast cancer incidence [10] and its competing mortality reliable.

Estimating the absolute risk of an event as accurately as possible is fundamental in clinical decision-making [25-28]. In the analysis of data in a competing risks setting, both results for the event of interest and its competing risks should be presented [29,30], and risk factors for each competing risk identified [29]. The necessity for analysis of both events of interest and their competing mortality can be explained through the definition of absolute cause-specific risk (given in Appendix).

Moreover, describing the natural history of cancer and possible pathways that an individual may go through is an essential component of any simulation model which is developed to assess the effectiveness of different intervention policies. In a microsimulation model, the life histories of many individuals are generated, and each history substantially depends on the value of different risk factors for a given individual. For example, a woman who has a relative with breast cancer is at a higher risk of being diagnosed with breast cancer at a younger age. Other competing events, such as death due to different causes can also be influenced by the risk factors. For example, a woman aged 70 or above may die due to heart attack before she is diagnosed with breast cancer. Therefore, the effect of risk factors on all competing events should be studied and described to model realistic life paths of individuals. The simulation models could be either developed for evaluating costs and benefits of cancer interventions at the population level, or for an individual according to her risk factors, as the practice of personalized medicine [31].

A cost-effective breast cancer screening policy relies on knowing the absolute risk of breast cancer, and as discussed, to obtain the absolute risk, competing risks should also be studied and taken into account. The absolute risk can then be used to decide whether a woman is categorized as high risk and whether a specific intervention with higher frequency should be recommended.

Gail et al. [32] developed an online risk assessment tool to estimate a woman’s absolute risk of developing invasive breast cancer [33]. In this tool, the absolute risk is estimated based on a woman’s risk factors including age, family history of breast cancer, number of biopsies, etc. A woman is identified at high risk if her lifetime risk of invasive breast cancer exceeds 20% [34]. Gail’s model [32] also investigates competing risks, namely the age-specific risk of mortality from causes other than breast cancer by using national mortality rates.

## Conclusions

In this study, we show that some risk factors are statistically significant for both the main event of interest (incidence of invasive breast cancer) and a competing event (mortality due to non-breast cancer causes). In addition, we identify other risk factors associated with all-cause mortality and cause-specific mortality. All these factors must be taken into account in estimating the probability of a competing event. With more accurate estimation of the probability of a competing event, the estimations of absolute risk of the main event of interest can be estimated more precisely. This in turn leads to more robust cost-effective analysis of preventive, diagnosis, and treatment policies which are decided based on the results of the absolute risk prediction model.

We used a cause-specific model to conduct regression analysis on our competing risks data. In addition, we fitted the Fine-Gray model and the results were very close to the results of the cause-specific hazard model. The cause-specific model is more clinically and biologically understandable since it directly describes the effect of a covariate on a specific cause and its hazard, regardless the effect of other covariates [15,30]. In our data, the risk of both breast cancer incidence [10] and its competing mortality are minor, for which the Cox cause-specific hazard is more reliable to be used [30]. Moreover, the generality of hazard of subdistribution is restricted to populations with similar characteristics and competing risk rate [35], the latter being relaxed in the cause-specific hazard model. On the other hand, the subdistribution hazard can be used to calculate the cumulative incidence function of the event of interest and to directly interpret the effect of covariates on this function [36].

## Appendix-Calculating absolute cause-specific risk

When there are *K* competing causes, the cause-specific hazard, which is defined as the instantaneous probability of failing from cause *k* is

(1) $$\lambda_{k}(t|Z)=\lim_{\Delta t\to 0}\frac{P(t\leq T\leq t+\Delta t,C=k|T\geq t,Z)}{\Delta t}\text{,}$$

where *Z* is a vector of covariates (risk factors), and the overall hazard is $\lambda (t|Z)=\sum_{k=1}^{K}\lambda_{k}(t|Z)$.The absolute cause-specific risk or cumulative incidence function (CIF) for cause *k* is defined as follows [37]:

(2) $$I_{k}(t|Z)=P(T\leq t,C=k)=\int_{o}^{t}\lambda_{k}(s|Z)S(s|Z)ds\text{.}$$

In equation (2), $S(t|Z)=P(T\geq t|Z)=e^{-\Lambda (t|Z)}$ is the overall survival function and $\Lambda (t|Z)=\int_{0}^{t}\lambda (s|Z)ds$ is the cumulative hazard. Therefore, the cumulative hazard is a function of all cause-specific hazards k=1,…,K. Equation (2) clearly shows that to obtain the cumulative risk of an event of interest (in our case, breast cancer incidence), the cause-specific hazard for all competing causes (competing mortality) should be also estimated.

## Competing interests

The authors report no conflicts of interest associated with this work.

## Authors’ contributions

ST prepared the CNBSS data for analysis, conducted the statistical analysis, and drafted the initial manuscript. JF helped in conducting the statistical analysis and writing the literature review of the manuscript. DB and NM contributed in conducting statistical methods, constructing the models, and interpreting the results. ABM designed, coordinated and directed the Canadian National Breast Screening Study (NBSS), and helped in understanding the data and interpreting the statistical results. BJH assisted in understanding the data and interpreting the statistical results. AJ and BJH are the co-PIs of the project and performed critical revisions of the manuscript. All authors read and approved the final manuscript.

## Pre-publication history

The pre-publication history for this paper can be accessed here:

http://www.biomedcentral.com/1471-2407/12/299/prepub
